# 3,3′-Dibromo-1,1′-[ethyl­enedioxy­bis(nitrilo­methyl­idyne)]dibenzene

**DOI:** 10.1107/S1600536808020692

**Published:** 2008-07-31

**Authors:** Wen-Kui Dong, Yu-Jie Ding, Ya-Ling Luo, Hai-Bo Yan, Li Wang

**Affiliations:** a School of Chemical and Biological Engineering, Lanzhou Jiaotong University, Lanzhou 730070, People’s Republic of China; b Department of Biochemical Engineering, Anhui University of Technology and Science, Wuhu 241000, People’s Republic of China

## Abstract

In the centrosymmetric title compound, C_16_H_14_Br_2_N_2_O_2_, the intra­molecular interplanar distance between the parallel benzene rings is 1.305 (3) Å, while the inter­molecular interplanar distance (between neighbouring mol­ecules) is 3.463 (3) Å, exhibiting obvious strong inter­molecular π–π stacking inter­actions.

## Related literature

For related literature, see: Akine *et al.* (2006 ▶); Atwood & Harvey (2001 ▶); Dong & Duan (2008 ▶); Dong *et al.* (2007 ▶); Dong, Duan *et al.* (2008 ▶); Dong, Shi *et al.* (2008 ▶); Katsuki (1995 ▶); Sun *et al.* (2004 ▶).
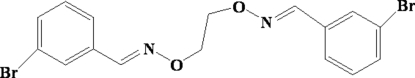


## Experimental

### 

#### Crystal data

C_16_H_14_Br_2_N_2_O_2_
*M* *_r_* = 426.11
Monoclinic, 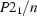

*a* = 4.5072 (7) Å
*b* = 7.615 (2) Å
*c* = 23.180 (3) Å
β = 93.523 (2)°
*V* = 794.1 (3) Å^3^
*Z* = 2
Mo *K*α radiation
μ = 5.11 mm^−1^
*T* = 298 (2) K
0.42 × 0.27 × 0.15 mm

#### Data collection

Siemens SMART 1000 CCD area-detector diffractometer
Absorption correction: multi-scan (*SADABS*; Sheldrick, 1996 ▶) *T*_min_ = 0.223, *T*_max_ = 0.514 (expected range = 0.201–0.464)
3840 measured reflections
1400 independent reflections
1168 reflections with *I* > 2σ(*I*)
*R*_int_ = 0.043

#### Refinement

*R*[*F* ^2^ > 2σ(*F* ^2^)] = 0.037
*wR*(*F* ^2^) = 0.097
*S* = 1.04
1400 reflections
100 parameters
H-atom parameters constrained
Δρ_max_ = 0.28 e Å^−3^
Δρ_min_ = −0.49 e Å^−3^

### 

Data collection: *SMART* (Siemens, 1996 ▶); cell refinement: *SAINT* (Siemens, 1996 ▶); data reduction: *SAINT*; program(s) used to solve structure: *SHELXS97* (Sheldrick, 2008 ▶); program(s) used to refine structure: *SHELXL97* (Sheldrick, 2008 ▶); molecular graphics: *SHELXTL* (Sheldrick, 2008 ▶); software used to prepare material for publication: *SHELXTL*.

## Supplementary Material

Crystal structure: contains datablocks global, I. DOI: 10.1107/S1600536808020692/ww2122sup1.cif

Structure factors: contains datablocks I. DOI: 10.1107/S1600536808020692/ww2122Isup2.hkl

Additional supplementary materials:  crystallographic information; 3D view; checkCIF report

## Figures and Tables

**Fig. 1.**
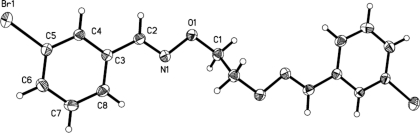
The molecular structure of the title compound with atom numbering scheme [Symmetry codes: -x - 1,-y,-z]. Displacement ellipsoids for non-hydrogen atoms are drawn at the 30% probability level.

## supplementary crystallographic information

### Comment

Schiff base compounds have played an important role in the development of coordination chemistry as they can readily form stable complexes with most metal ions (Dong & Duan, 2008; Dong, Duan *et al.*, 2008; Dong, Shi *et al.*, 2008; Atwood & Harvey, 2001). These complexes are very interesting in many fields, such as catalysis and enzymatic reactions (Akine *et al.*, 2006), magnetism and molecular architectures (Sun *et al.*, 2004; Katsuki, 1995). However, to the best of our knowledge, the complexes derived from the Schiff base 3,3'-dibromo-1,1'-[ethylenedioxybis(nitriloethylidyne)]dibenzene have never been reported so far. Information on the structure of the Schiff base compound will help us understand the interactions in the molecule so as to further design and synthesize complexes derived from this ligand. As a further investigation on such compounds, we report herein the synthesis and crystal structure of the Schiff base bisoxime compound 3,3'-dibromo-1,1'-[ethylenedioxybis(nitriloethylidyne)]dibenzene, shown in Fig. 1.

The X-ray crystallography reveals the title compound crystallizes in the monoclinic system, space group P2(1)/n with a = 4.5072 (7) Å, b = 7.615 (2) Å, c = 23.180 (3) Å, β = 93.523 (2) ° and *Z*= 2. The structure of the title compound consists of discrete C_16_H_14_Br_2_N_2_O_2_ molecules in which all bond lengths are in normal ranges.

The molecule is disposed about a crystallographic centre of symmetry at the mid-point of the (CH_2_—CH_2_) linkage adopting an anti-symmetrized conformation in which two benzaldoxime moieties adopt an extended form. Both intra- and inter-molecular hydrogen bonds are not observed in the title compound. The intramolecular plane-to-plane distance of the benzene rings was found to be 1.305 Å, while that of the intermolecular plane-to-plane distance (between neighbouring molecules) was found to be 3.463 (3) Å, exhibiting obvious strong intermolecular π-π stacking interactions.

### Experimental

3,3'-Dibromo-1,1'-[ethylenedioxybis(nitriloethylidyne)]dibenzene was synthesized according to our previous work (Dong *et al.*, 2007). To an ethanol solution (3 ml) of 3-bromo-benzaldehyde (398.7 mg, 2.15 mmol) was added dropwise an ethanol solution (2 ml) of 1,2-bis(aminooxy)ethane (96.2 mg, 1.04 mmol). The mixture solution was stirred at 328 K for 4 h. After cooling to room temperature, the precipitate was filtered off, and washed successively with ethanol and ethanol-hexane mixture (1:4), respectively. The product was dried *in vacuo* to yield 366.0 mg (Yield, 82.3%) of colorless microcrystals; m.p. 363.5 - 365.5 K. Anal. Calcd. for C_16_H_14_Br_2_N_2_O_2_: C, 45.10; H, 3.31; N, 6.57. Found: C, 45.01; H, 3.20; N, 6.43%.

Single crystals were obtained by slow evaporation from a ethanol-acetone mixed solution of the title compound at room temperature.

### Refinement

Non-H atoms were refined anisotropically. H atoms were treated as riding atoms with distances C—H = 0.97 (CH_2_), 0.93 Å (CH), and *U*_iso_(H) = 1.2 *U*_eq_(C).

### Crystal data

**Table:**

C_16_H_14_Br_2_N_2_O_2_	*F*_000_ = 420
*M**_r_* = 426.11	*D*_x_ = 1.782 Mg m^−^^3^
Monoclinic, *P*2_1_/*n*	Mo *K*α radiation λ = 0.71073 Å
Hall symbol: -P 2yn	Cell parameters from 2000 reflections
*a* = 4.5072 (7) Å	θ = 2.6–26.7º
*b* = 7.615 (2) Å	µ = 5.11 mm^−^^1^
*c* = 23.180 (3) Å	*T* = 298 (2) K
β = 93.523 (2)º	Needle-shaped, colorless
*V* = 794.1 (3) Å^3^	0.42 × 0.27 × 0.15 mm
*Z* = 2	

### Data collection

**Table:**

Siemens SMART 1000 CCD area-detector diffractometer	1400 independent reflections
Radiation source: fine-focus sealed tube	1168 reflections with *I* > 2σ(*I*)
Monochromator: graphite	*R*_int_ = 0.043
*T* = 298(2) K	θ_max_ = 25.0º
ω and φ scans	θ_min_ = 1.8º
Absorption correction: multi-scan(SADABS; Sheldrick, 1996)	*h* = −5→5
*T*_min_ = 0.223, *T*_max_ = 0.514	*k* = −8→9
3840 measured reflections	*l* = −19→27

### Refinement

**Table:**

Refinement on *F*^2^	Secondary atom site location: difference Fourier map
Least-squares matrix: full	Hydrogen site location: inferred from neighbouring sites
*R*[*F*^2^ > 2σ(*F*^2^)] = 0.037	H-atom parameters constrained
*wR*(*F*^2^) = 0.097	*w* = 1/[σ^2^(*F*_o_^2^) + (0.0535*P*)^2^ + 0.3108*P*] where *P* = (*F*_o_^2^ + 2*F*_c_^2^)/3
*S* = 1.04	(Δ/σ)_max_ < 0.001
1400 reflections	Δρ_max_ = 0.28 e Å^−^^3^
100 parameters	Δρ_min_ = −0.49 e Å^−^^3^
Primary atom site location: structure-invariant direct methods	Extinction correction: none

### Special details

**Table:**

Geometry. All e.s.d.'s (except the e.s.d. in the dihedral angle between two l.s. planes) are estimated using the full covariance matrix. The cell e.s.d.'s are taken into account individually in the estimation of e.s.d.'s in distances, angles and torsion angles; correlations between e.s.d.'s in cell parameters are only used when they are defined by crystal symmetry. An approximate (isotropic) treatment of cell e.s.d.'s is used for estimating e.s.d.'s involving l.s. planes.
Refinement. Refinement of *F*^2^ against ALL reflections. The weighted *R*-factor *wR* and goodness of fit *S* are based on *F*^2^, conventional *R*-factors *R* are based on *F*, with *F* set to zero for negative *F*^2^. The threshold expression of *F*^2^ > σ(*F*^2^) is used only for calculating *R*-factors(gt) *etc*. and is not relevant to the choice of reflections for refinement. *R*-factors based on *F*^2^ are statistically about twice as large as those based on *F*, and *R*- factors based on ALL data will be even larger.

### Fractional atomic coordinates and isotropic or equivalent isotropic displacement parameters (Å^2^)

**Table:**

	*x*	*y*	*z*	*U*_iso_*/*U*_eq_	
Br1	0.56056 (10)	0.86904 (5)	0.160074 (19)	0.0576 (2)	
N1	−0.2668 (6)	0.2442 (4)	0.05861 (13)	0.0381 (7)	
O1	−0.4716 (6)	0.2385 (3)	0.00969 (11)	0.0422 (6)	
C1	−0.6114 (8)	0.0710 (5)	0.00676 (17)	0.0396 (8)	
H1A	−0.7723	0.0722	−0.0230	0.048*	
H1B	−0.6952	0.0452	0.0434	0.048*	
C2	−0.1591 (8)	0.3960 (4)	0.06447 (16)	0.0373 (8)	
H2	−0.2198	0.4837	0.0384	0.045*	
C3	0.0595 (7)	0.4362 (4)	0.11157 (15)	0.0324 (8)	
C4	0.1868 (8)	0.6001 (4)	0.11401 (15)	0.0350 (8)	
H4	0.1337	0.6827	0.0857	0.042*	
C5	0.3929 (8)	0.6427 (4)	0.15821 (16)	0.0369 (8)	
C6	0.4796 (9)	0.5227 (5)	0.19990 (16)	0.0449 (9)	
H6	0.6198	0.5523	0.2294	0.054*	
C7	0.3545 (9)	0.3565 (5)	0.19728 (18)	0.0485 (10)	
H7	0.4098	0.2741	0.2256	0.058*	
C8	0.1504 (9)	0.3121 (5)	0.15356 (16)	0.0412 (9)	
H8	0.0719	0.1992	0.1517	0.049*	

### Atomic displacement parameters (Å^2^)

**Table:**

	*U*^11^	*U*^22^	*U*^33^	*U*^12^	*U*^13^	*U*^23^
Br1	0.0701 (4)	0.0416 (3)	0.0597 (3)	−0.0158 (2)	−0.0076 (2)	−0.00700 (18)
N1	0.0374 (16)	0.0381 (17)	0.0381 (18)	−0.0031 (13)	−0.0038 (13)	−0.0018 (13)
O1	0.0438 (14)	0.0346 (13)	0.0468 (15)	−0.0078 (11)	−0.0083 (12)	0.0016 (11)
C1	0.037 (2)	0.0389 (19)	0.043 (2)	−0.0032 (16)	−0.0004 (16)	−0.0033 (16)
C2	0.0387 (19)	0.0329 (19)	0.040 (2)	0.0004 (15)	0.0022 (16)	0.0032 (15)
C3	0.0310 (18)	0.0343 (18)	0.0323 (19)	0.0008 (14)	0.0061 (14)	−0.0021 (14)
C4	0.039 (2)	0.0322 (18)	0.0337 (19)	0.0024 (15)	0.0026 (15)	−0.0001 (14)
C5	0.038 (2)	0.0344 (19)	0.038 (2)	−0.0041 (15)	0.0036 (16)	−0.0037 (15)
C6	0.047 (2)	0.049 (2)	0.037 (2)	0.0008 (18)	−0.0054 (18)	−0.0027 (17)
C7	0.052 (2)	0.046 (2)	0.047 (2)	0.0059 (19)	−0.0024 (19)	0.0120 (18)
C8	0.048 (2)	0.0334 (19)	0.043 (2)	−0.0055 (17)	0.0102 (18)	0.0027 (16)

### Geometric parameters (Å, °)

**Table:**

Br1—C5	1.881 (3)	C3—C8	1.399 (5)
N1—C2	1.258 (4)	C4—C5	1.379 (5)
N1—O1	1.418 (4)	C4—H4	0.9300
O1—C1	1.423 (4)	C5—C6	1.369 (5)
C1—C1^i^	1.521 (7)	C6—C7	1.386 (5)
C1—H1A	0.9700	C6—H6	0.9300
C1—H1B	0.9700	C7—C8	1.368 (5)
C2—C3	1.457 (5)	C7—H7	0.9300
C2—H2	0.9300	C8—H8	0.9300
C3—C4	1.373 (5)		
			
C2—N1—O1	110.0 (3)	C3—C4—H4	119.8
N1—O1—C1	109.3 (3)	C5—C4—H4	119.8
O1—C1—C1^i^	110.5 (4)	C6—C5—C4	121.1 (3)
O1—C1—H1A	109.5	C6—C5—Br1	119.9 (3)
C1^i^—C1—H1A	109.5	C4—C5—Br1	119.0 (3)
O1—C1—H1B	109.5	C5—C6—C7	118.8 (3)
C1^i^—C1—H1B	109.5	C5—C6—H6	120.6
H1A—C1—H1B	108.1	C7—C6—H6	120.6
N1—C2—C3	120.8 (3)	C8—C7—C6	120.7 (4)
N1—C2—H2	119.6	C8—C7—H7	119.6
C3—C2—H2	119.6	C6—C7—H7	119.6
C4—C3—C8	118.7 (3)	C7—C8—C3	120.2 (4)
C4—C3—C2	118.9 (3)	C7—C8—H8	119.9
C8—C3—C2	122.3 (3)	C3—C8—H8	119.9
C3—C4—C5	120.4 (3)		
			
C2—N1—O1—C1	−174.7 (3)	C3—C4—C5—Br1	−179.8 (3)
N1—O1—C1—C1^i^	−67.7 (5)	C4—C5—C6—C7	−0.5 (6)
O1—N1—C2—C3	−179.6 (3)	Br1—C5—C6—C7	−179.5 (3)
N1—C2—C3—C4	175.5 (3)	C5—C6—C7—C8	0.7 (6)
N1—C2—C3—C8	−2.8 (5)	C6—C7—C8—C3	−1.6 (6)
C8—C3—C4—C5	−2.1 (5)	C4—C3—C8—C7	2.3 (6)
C2—C3—C4—C5	179.6 (3)	C2—C3—C8—C7	−179.4 (4)
C3—C4—C5—C6	1.2 (5)		

Symmetry codes: (i) −*x*−1, −*y*, −*z*.
